# Career unreadiness in relation to anxiety and authoritarian parenting among undergraduates

**DOI:** 10.1080/02673843.2014.928784

**Published:** 2014-08-08

**Authors:** Chau-Kiu Cheung, Hoi Yan Cheung, Joseph Wu

**Affiliations:** ^a^ Department of Applied Social Studies, City University of Hong Kong, Kowloon, Hong Kong; ^b^ Faculty of Education, University of Macau, Macau, China

**Keywords:** career unreadiness, authoritarian parenting, anxiety

## Abstract

Career unreadiness, covering career indecision and career myth, is an issue for universities to address. Supposedly, career unreadiness is responsible for the university student's anxiety and partly results from authoritarian parenting during the student's childhood. This is an uncharted concern for this study to clarify. The study surveyed 229 undergraduates in two universities in Hong Kong, China. It employed structural equation modelling to clarify nexuses among career unreadiness, authoritarian parenting and anxiety, after minimising their measurement errors. Career unreadiness mediated the negative effect of authoritarian parenting on anxiety. Nevertheless, authoritarian parenting still maintained a negative direct effect on anxiety, after controlling for career unreadiness. The findings imply that reducing undergraduates' career unreadiness is justifiable to prevent their anxiety. Such a reduction would benefit from neutralising the demands of authoritarian parenting. More fundamentally, diverting authoritarian parenting is advisable.

A university is an institution to prepare its students for their careers (Gutman & Schoon, 2012; Pascarella & Terenzini, 2005) by giving them ample exposure to training, counselling, talks, internship, and many activities for honing their career readiness. Nevertheless, these activities are unlikely to be helpful to every student, and some students are concerned to find themselves unready for their careers (Gutman & Schoon, 2012). In this context, career unreadiness, as defined from the perspective of the undergraduate, refers to a difficulty in forming decisions about and beliefs in an enduring vocational commitment (Osipow & Gati, 1998). The undergraduate's career unreadiness tends to be an instance of emergent adulthood during which the undergraduate postpones or prolongs his or her career development (Arnett, 2007). This is why career counselling is a prominent service for the undergraduate (Gati, Houminer, & Fassa, 1997). Crucially, the undergraduate can be unready for the career, even though he or she achieves romantic and family support (Nelson & Barry, 2005). Career unreadiness is undesirable, because of its provocation of anxiety, in the view of behavioural inhibition theory (McNaughton & Gray, 2000; Neal, Edelman, & Glachan, 2002). Herewith, anxiety means a feeling about one's inadequacy to meet challenges in life (McDougall, DeWit, King, Miller, & Killip, 2004; Parade, Leekes, & Blankson, 2010). Moreover, the theory envisions that both the student's career unreadiness and anxiety partly originate from the authoritarian parenting during the student's childhood. In this case, authoritarian parenting means the parent controls, dominates, or exerts demands on the child without showing warmth or support (Koumoundourou, Tsaousis, & Kounenou, 2011).

Career unreadiness is likely to be a link that connects both authoritarian parenting and anxiety. As this link has been identified as the gap in research, it is the focus of this study of university students to furnish empirical evidence. Essentially, career unreadiness, anxiety, and authoritarian parenting are all crucial factors in existing research and practice, and their nexuses are especially of concern. The study thus aims to show the important role of career unreadiness as a mediator between authoritarian parenting and anxiety. For this aim, the study identified the latent factors in an integrated model through structural equation modelling, and such a sophisticated approach is largely missing in existing studies (e.g. Chan & Koo, 2010; Kelly & Lee, 2002; Snatos & Coimbra, 2000; Strauser, Lustig, Cogdal, & Uruk, 2006). In addition, the role of career unreadiness is worth clarification, in view of alternative views implying that it is normal or even that it leads to beneficial exploration (Arnett, 2007; Nelson & Barry, 2005; Super, 1990).

Career readiness is desirable before engaging in a career (Gati, Krausz, & Osipow, 1996). The unreadiness is not a singular concept, but includes distinct components, notably indecision and myth (Osipow & Gati, 1998). Career indecision refers to the individual's decisional or behavioural actions pertaining to difficulty and avoidance in making decisions and commitments. Career myth refers to the cognitive component involved in the exaggeration of the ideals and obligations of a career. These two components have their respective meanings. Career indecision is clearly associated with confusion, conflict, and anxiety (Strauser et al., 2006). This indecision also denotes falling into the mire of exploration (Leung, Hou, Gati, & Li, 2011), an example of which is that of the young person's inadequate ego development (Koumoundourou et al., 2011). By contrast, career myth implies inadequate exploration and acquisition of practical career knowledge. This inadequacy, in turn, originates from the individual's dependence on his or her family (Hardin, Varghese, Tran, & Carlson, 2006). Accordingly, a family or parents that are controlling or authoritarian may disrupt the young person's career exploration and acquisition of knowledge to debunk career myths (Dietrich & Kracke, 2009). Career indecision and myth are distinguishable in that the former is motivational and the latter is cognitive, and as such they may have distinctive connections to anxiety and authoritarian parenting.

Anxiety is a state wherein an individual perceives in one's self an inadequacy to meet societal challenges (Spielberger, 1983). Thus, feeling inadequate, insecure, ill-equipped, and unconfident are common manifestations of anxiety. It is a mood rather than a clinical disorder, and it is chartable in this study. Anxiety also arises from protean social change, which requires the young person's exploration and adaptation to the environment (Taimalu, Lahikainen, Korhonen, & Kraav, 2006). This exploration and adaptation, in terms of behaviour and cognition, are likely to prevent anxiety (Marcussen, 2006). Anxiety is of crucial concern because of its interference with psychosocial development (Turiel, 2006). Accordingly, anxiety signifies irrationality and mental conflict that could undermine the young person's engagement in general daily and developmental tasks.

Authoritarian parenting, as perceived by the young person, refers to parents' domination, control, regulation, and overestimation in a strict and unreasonable manner (Buri, 1991); an example is the prohibition of the young person's questioning of parental decisions. Authoritarian parenting, as perceived by the young person is consequential and noteworthy (Bolkan, Sano, de Costa, Acock, & Day, 2010), since it is likely to weaken trust in and attachment to the young person's parents (Bell, 2009). This parenting style is prevalent, particularly in China and Hong Kong (Lai, Zhang, & Wan, 2000). Hong Kong is therefore a relevant site for this study of authoritarian parenting in relation to career unreadiness and anxiety.

## Relating career unreadiness to anxiety and authoritarian parenting

Behavioural inhibition theory is capable of providing grounds for the nexuses among authoritarian parenting, career unreadiness, and anxiety experienced by the university student. The theory rests on ample physiological and psychological evidence about the workings of the behavioural inhibition system in the person's mind and body (McNaughton & Gray, 2000; Neal et al., 2002). The system maintains one's sensitivity to behavioural inhibition from the outside, such as parental punishment, and then drives one's behavioural inhibition as a reaction (Degnan & Fox, 2007; McNaughton & Gray, 2000). Such a reaction involves a number of physiological indicators, including an increased heart rate, dilation of the pupils, salivary cortisol, and vocal tension. Behavioural inhibition imposed from the outside and invoked in the body operates to retard one's activity, including exploration, socialising, verbalising, and even smiling. This is a case of latency, which can generate conflict, fear, and suffering. In an attempt to resolve conflict, one raises arousal, attention, and thus tension, which are symptoms of anxiety. Hence, behavioural inhibition theory envisions behavioural inhibition as a mechanism thwarting one's social development and thus provoking one's anxiety. Specifically, the theory underpins the following hypotheses.

The basic and general hypotheses are that a young person's career unreadiness, indecision or myth, sustains anxiety and stems from his or her authoritarian upbringing. These hypotheses rest on theory and research about relationships between unreadiness, anxiety, and authoritarian parenting (Koumoundourou et al., 2011; Meyer-Griffith, Reardon, & Hartley, 2009; Strauser et al., 2006). According to behavioural inhibition theory, career unreadiness is likely to translate the impact of authoritarian parenting into anxiety (Degnan & Fox, 2007; McNaughton & Gray, 2000; Neal et al., 2002). Particularly, the theory suggests that an individual becomes anxious in response to the entrenched behavioural inhibition experience. The experience involves intrusive or authoritarian parenting, which reaffirms a system formed by physiological and dispositional bases. For instance, the authoritarian parenting increases the child's heart rate and reticence, especially when facing strangers. The system of behavioural inhibition then maintains latency in the child, undermining the child's sociability, approachability, and exploration of the environment. Furthermore, this system keeps the child in a state of arousal, attention, and, ultimately, anxiety.

According to behavioural inhibition theory, anxiety results from latency or the lack of exploration of the environment, which can be represented by career indecision (Germeijs, Verschueren, & Soenens, 2006; Neal et al., 2002). That is, career indecision is likely to generate a feeling of anxiety (Hypothesis 1). Similarly, myth that one is incapable is likely to make one anxious (Davis & Valentiner, 2000). The anxiety effect happens when career development is set as a task required by the university and society that hinges on exploration of the work environment (Hirschi, 2009). Thus career myth may inhibit one's self-efficacy belief about one's career. Self-efficacy belief appears to be a determinant of exploration into career development and the exploration tends to prevent anxiety (Gati, Houminer, & Fassa, 1997; Lent, Brown, & Hackett, 2000). In this way, career myth is likely to foment anxiety. This behavioural-inhibiting mechanism is thus consistent with the role played by self-efficacy. In this respect, anxiety is a state of mind, whereas career unreadiness consists of experience, action, decision, and beliefs that are likely to have happened earlier and taken a longer time to develop. It reflects the idea that an earlier and more stable experience affects a momentary feeling (Davis, 1985). Specifically, anxiety is likely to be an emotional response to cognition, motivation, and learning from experience or action (Bouton, Mineka, & Barlow, 2001). Research has shown that hopelessness, negative thoughts, and inability to solve problems are precursors to an individual's anxiety (Eremsoy, Celimli, & Gencoz, 2005). Thus, career indecision and myth, albeit different, are both likely to be the behavioural and cognitive determinants of anxiety. Research has shown that career indecision in particular is conducive to anxiety (Kelly & Lee, 2002).

A young person's career unreadiness, indecision or myth, is also likely to stem from authoritarian parenting, which the young person experienced growing up (Hypothesis 2). In general, authoritarian parenting represents an external force that maintains behavioural inhibition, according to behavioural inhibition theory (Neal et al., 2002; Tokar, Withrow, Hull, & Moradi, 2003). This inhibitive influence has appeared in some existing studies as well (Bryant, Zvonkovic, & Reynolds, 2006; Tokar et al., 2003). In this regard, theory and research hold that career readiness results from secure and encouraging parenting, which fosters a sense of freedom in the young person to aspire, explore, and decide their choice of career. Such parenting would involve responsiveness to and support for the young person's autonomy, security, and independence from the parents. These conditions disappear in the face of authoritarian parenting, which impedes the parent's responsiveness and support. This theory is also consistent with the view that authoritarian parenting hinders the young person's self-efficacy belief, which in turn leads to the young person's career unreadiness, indecision or myth (Betz, Klein, & Taylor, 1996; Lease & Dahlbeck, 2009). In support of the theory, research has shown that authoritarian parenting results in career indecision and deters career exploration on the part of the young person (Koumoundourou et al., 2011). However, some studies fail to demonstrate the expected effects of authoritarian parenting on career unreadiness, including indecision and inadequate exploration (Vignoli, 2009). These mixed findings present a rationale for the present investigation, with the identification of latent factors through structural equation modelling.

Another likelihood is that the current anxiety springs from the authoritarian parenting that has happened earlier in the young person's life (Hypothesis 3) as interpreted using behavioural inhibition theory and previous studies (Beran & Violato, 2004; Degnan & Fox, 2007). According to the theory, authoritarian parenting is a social force that inhibits the young person's own drive and realisation of talent. This theory implies that the young person has his or her drives and talents, and wishes to realise them, and will suffer from anxiety if such wishes fail to meet the challenges of authoritarian parenting. Conversely, supportive and warm parenting will likely prevent the young person's anxiety (Leung, Wong, Wong, & McBridge-Chang, 2010). Due to the possible influence of authoritarian parenting on anxiety, authoritarian parenting is an important control factor that will clarify the net effect of career unreadiness on anxiety. Meanwhile, career unreadiness is likely to enact a mediating role between authoritarian parenting and anxiety.

## Hypothesis testing

A recapitulation of the above discussion maintains the following key hypotheses regarding the young person or undergraduate:

1. Career unreadiness (indecision and myth) shows a positive effect on anxiety.
2. Career unreadiness (indecision and myth) receives a positive effect from authoritarian parenting.
3. Authoritarian parenting gives a positive effect on anxiety.

This series of hypotheses suggests that career unreadiness mediates the effect of authoritarian parenting on anxiety. That is, authoritarian parenting supposedly exerts an indirect effect on anxiety through mediation by career indecision and myth. Based on the realist philosophy, a modelling approach seeks to examine whether the hypothesised effects fit the reality (Hunt, 1994; Layder, 1990). The philosophy regards the fit with the commonly unobservable reality as paramount and endorses the theoretically based model that realises the fit. Essentially, the philosophy rejects alternative models that fit data but lack theoretical grounds (Lewis, 2000; Stelzl, 1986). Realist modelling thus focuses on a true model, rather than trial and error. The model involved also needs to minimise problems with measurement error and confounding by background and methodological factors. This model serves to identify anxiety, career unreadiness, and authoritarian factors while being free of measurement and mythological errors. The latter refers to a common method bias resulting from the use of rating scales (Podsakoff, MacKenzie, & Podsakoff, 2003). In addition, the modelling controls the basic background factors of gender, age, and the year of study in the university. Among these factors, age and female gender tend to raise anxiety, whereas the year of study appears to reduce anxiety (Leung et al., 2010). In contrast, age and male gender, and the year of study tend to diminish career unreadiness (Turner & Lapan, 2002). Controlled for the year for study, age can also serve as a proxy for work experience, as the older students would have more work experience before starting their university study. Notably, work experience can be a confounder, when it may affect career unreadiness and anxiety (Creed, 1999; Flouri & Buchanan, 2003).

## Method

A survey of 229 undergraduates from two universities in Hong Kong, China, provided the data for the study. Their participation in the survey was voluntary and fulfilled the requirement approved by the institutional ethical review board. The self-administered survey took place in classrooms to ensure that the students completed the questionnaire independently.

At the time of the survey, that is prior to 2012, universities in Hong Kong typically offered three-year undergraduate programmes. Correspondingly, the respondents included 63 year-1 students, 102 year-2 students, and 64 year-3 students. Their average year of study was 2.0 years, and their average age was 20.9 years (with a range from 18 to 26 years). Among the respondents, 57.0% were females.

### Measurement

The survey questionnaire measured career unreadiness, anxiety, and authoritarian parenting by using multiple items with rating scales. Originally, the items had a five-point Likert scale for measuring both anxiety and authoritarian parenting, and a nine-point rating scale for measuring career unreadiness. A scoring procedure scored every item on a 0–100 scale, with the lowest point scored 0, highest point scored 100, whereas the three intermediate points scored 25, 50, and 75, respectively, to facilitate description and comparison, and avoid confusion in examining the original five-point and nine-point scales (Preston & Colman, 2000; Stewart & Ware, 1993).

Career unreadiness comprised four items to measure career indecision and three items to measure career myth, adapted from the Career Decision-making Difficulties Questionnaire (Osipow & Gati, 1998). Sample items included ‘a general fear of failure’ for career indecision and ‘a belief that entering a career will solve personal problems’ for career myth (see Table 1). Composite reliability was 0.749 and 0.727 for career indecision and myth, respectively.

**Table 1:** Standardised factor loadings, based on Model 1.

Factor/item	Trait	Method
Authoritarian parenting (When I was growing up)		
Expecting me to do it immediately without asking any questions	0.640	0.063
Not allowing me to question any decision	0.592	0.087
Feeling that more force should be used	0.604	0.082
Teaching me who was boss in the family	0.526	0.165
Getting very upset if I tried to disagree with her/him	0.591	− 0.013
Punishing me if I did not meet those expectations	0.539	0.014
Strictly and forcibly dealing with me	0.346	0.204
Telling me exactly what she/he wanted me to do	0.415	0.229
Insisting that I conformed to those expectations	0.431	0.134
Career indecision		
A general difficulty in making decisions	0.790	0.049
A general need for confirmation and support for decisions	0.682	0.133
A general tendency to avoid commitment	0.476	0.087
A general fear of failure	0.633	0.142
Career myth		
A belief that entering a career will solve personal problems	0.721	0.120
A belief that there is an ideal career which can fulfil all aspirations	0.507	0.225
A belief that a career choice is a one-time thing	0.804	0.243
Anxiety		
I feel nervous and restless	0.579	− 0.200
I feel satisfied with myself (r)	0.379	− 0.549
I wish I could be as happy as others seem to be	0.360	− 0.117
I feel like a failure	0.550	− 0.035
I feel that difficulties are piling up so that I cannot overcome them	0.584	− 0.059
I worry too much over something that really does not matter	0.510	− 0.009
I am happy (r)	0.396	− 0.558
I have disturbing thoughts	0.520	− 0.004
I lack self-confidence	0.427	− 0.084
I feel secure (r)	0.407	− 0.551
I feel inadequate	0.494	− 0.059
Some unimportant thoughts run through my mind and bother me	0.550	− 0.014
I take disappointments so keenly that I cannot put them out of my mind	0.660	− 0.051
I get in a state of tension or turmoil as I think over my recent concerns	0.491	− 0.015

Note: (r): reverse scoring.

The measure for anxiety involved 14 items, including three positively phrased items, which required a reversal of scoring, as adapted from the Anxiety Inventory (Spielberger, 1983). Sample items included were ‘I feel inadequate’ for negatively phrased items and ‘I am happy’ for positively phrased items (see Table 1). They referred to the respondent's feelings at the time of the survey. The composite reliability of anxiety was 0.876.

Authoritarian parenting involved nine items pertaining to the parenting practice that the respondent received during growing up, as adapted from the Parental Style Scale (Buri, 1991). A sample item was ‘as I was growing up, my mother/father did not allow me to question any decision she/he had made’. The composite reliability of authoritarian parenting was 0.797. Notably, authoritarian parenting was a childhood experience recalled by the student.

### Analytic procedure

**Figure 1:**
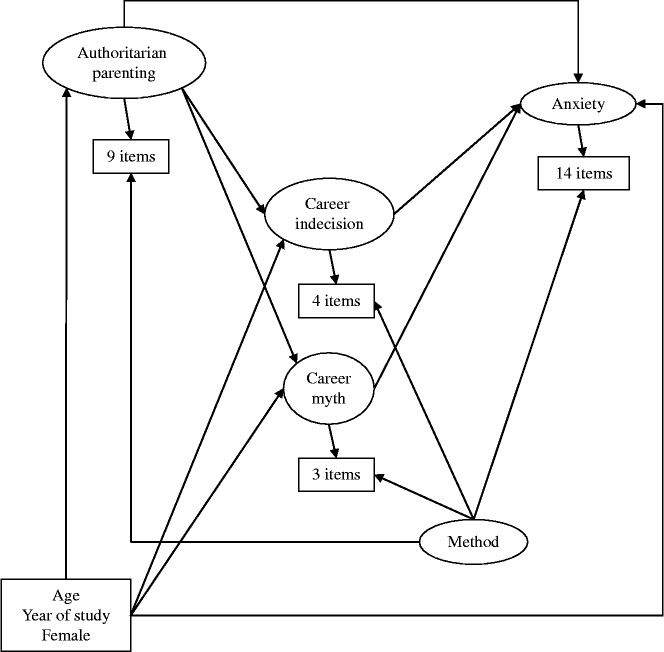
Schema of the basic model.

Structural equation modelling enabled a holistic or integrated examination of the connections among career unreadiness, anxiety, and authoritarian parenting to minimise confusion and any problems with measurement error (Muthen & Muthen, 2006). The minimising of measurement error depended on a confirmatory factor analytic part of the model to specify items, in order to identify the true factors of career unreadiness, anxiety, and authoritarian parenting, with the aid of a method factor (see Figure 1). Representing a mutlitrait-monomethod approach of measurement, this part identified the trait factor of career indecision using its four items, career myth using its three items, anxiety using its 14 items, and authoritarian parenting using its nine items (Cole, 1987). The modelling specified all items to load with equal (metric) magnitude on the method factor, while constrained to be independent of the four trait factors (Podsakoff et al., 2003). The four identified trait factors, but not the method factor, were thus useful for recovering their connections in the structural relation part of the modelling. This part involved a recursive model running from background factors to anxiety, through mediation by authoritarian parenting and career unreadiness successively (see Figure 1). In this regard, the model estimated total and indirect or mediated effects, as well as direct effects (MacKinnon, 2008).

The modelling fitted a basic model and an alternative model. Whereas the basic model (Model 1) treated career indecision and career myth as separate, the alternative model identified a factor to represent both the indecision and myth. Comparison of the fits of the two models showed whether the indecision and myth were separate or functionally equivalent indicators of career unreadiness. In this case, when the indecision and myth were not highly related, the basic model was preferable. Otherwise, if the indecision and myth were highly related, the alternative model would be preferable. Notably, as both models already fitted all relationships among the factors (i.e. career unreadiness, anxiety, and authoritarian parenting), they must give the best fit.

## Results

Simple descriptive statistics revealed that anxiety, career indecision, career myth, and authoritarian parenting were all at the mid-level for the undergraduates, on average (*M* = 49.8 to 55.7). This finding indicated no problems due to skewness in the distribution, and the variables were suitable for structural equation modelling (Kline, 2005). In addition, the finding showed that neither anxiety nor career unreadiness was a serious problem among the undergraduates.

Structural equation modelling was successful in identifying the models with good fit. First, the basic model (Model 1) achieved a very good fit (*L* ^2^(439) = 494, *p* = 0.036, BIC = 64,120, SRMR = 0.053, RMSEA = 0.023, CFI = 0.971), notably with a very high comparative goodness-of-fit index (see Table 2), where the CFI was equal to 0.971 and was greater than the good-fit threshold of 0.95 (Marsh, Hau, & Wen, 2004). Second, the alternative model (Model 2) also did not attain a good fit (*L* ^2^(456) = 662, *p* = 0.000, BIC = 64,228, SRMR = 0.062, RMSEA = 0.044, CFI = 0.900). The goodness-of-fit indicators showed the inadequacy of regarding career indecision and career myth as the equivalent indicators of career unreadiness. Moreover, comparison of the fits of the two models favoured the basic model, in view of its lower Bayesian information criterion (BIC = 64,120 vs. 64,228) and root-mean-square error of approximation (RMSEA = 0.023 vs. 0.044). Notably, alternative models that permutated the factors in other ways proved to yield the same fit (Stelzl, 1986). This meant that theoretical grounds were pivotal for using Model 1 for testing the hypotheses and showing other findings.

**Table 2:** Standardised direct effects.

Predictor	Model 1
Predicting anxiety	
Career indecision	0.604^*^^*^^*^
Career myth	0.150^*^
Authoritarian parenting	0.330^*^^*^^*^
Year of study	0.078
Age	− 0.075
Female	− 0.027
*R*^2^	0.510
Predicting career indecision	
Authoritarian parenting	0.196^*^^*^
Year of study	− 0.170
Age	0.111
Female	0.140
*R*^2^	0.067
Predicting career myth	
Authoritarian parenting	0.258^*^^*^^*^
Year of study	− 0.103
Age	0.032
Female	− 0.074
*R*^2^	0.083

^*^ *p* <  0.05, ^*^^*^ *p* <  0.01, ^*^^*^^*^ *p* <  0.001.

Findings from Model 1 showed that identification of the four trait factors was adequate. Items showed substantial loadings (λ > 0.30) on the trait factors in the presence of a method factor (see Table 1). This finding indicated the convergence of items in identifying their respective trait factors, and the discrimination among all the factors, including trait and method factors. Notably, the two trait factors of career indecision and myth were distinguishable from one another, given that their residual correlation was only 0.071. Nevertheless, some substantial standardised loadings on the method factor revealed a common method bias. Hence, the method factor was required to distil the trait factors that were free of measurement and other common method errors. With their separation from measurement and common method errors, the four trait factors were then eligible for an examination for their structural relations, as explained in detail below.

Generally, anxiety received the significant positive effects of career indecision, career myth, and authoritarian parenting (see Table 2). This finding lent support to Hypotheses 1 and 3 regarding the influences of career unreadiness and authoritarian parenting on anxiety. All the findings resulted from the control for background characteristics, and held similarly for both the male and female.

The apparent differential between the effect of career indecision (β = 0.604, see Table 2) and the effect of career myth (β = 0.150) on anxiety turned out to be statistically insignificant (*ΔL* ^2^ = 3, Δdf = 1, *p* = 0.083). Similarly, the apparent differential between the effects of authoritarian parenting on career indecision (β = 0.196) and career myth (β = 0.258) was statistically significant (*ΔL* ^2^ = 0, Δdf = 1, *p* = 1.000).

Career indecision consistently received a significant positive effect from authoritarian parenting (β = 0.196, see Table 2). This finding delivered partial support to Hypothesis 2. Moreover, the effect was significant for both male and female respondents (β = 0.318 and 0.173, respectively). In addition, no other significant effects on career indecision were present.

Career myth received a significant effect (β = 0.258) from authoritarian parenting in general (see Table 2), providing partial support to Hypothesis 2. In addition, the year of study in university manifested a significant negative effect on career myth only in males (β = 0.526).

With the mediation by the three factors of career unreadiness, authoritarian parenting showed a significant positive total effect on anxiety (β = 0.479, *p* <  0.001). The indirect or mediated effect (β = 0.148, *p* <  0.01) was also significant, as it revealed the mediation by the factors of career unreadiness. As such, the mediation accounted for nearly one-third of the total effect. Meanwhile, the effect of authoritarian parenting was not attributable to the gender difference, as the gender parameter made no significant difference in the parenting factor (β = − 0.046).

## Discussion

Some support for all the three hypotheses evolved from the structural equation modelling using data collected from the undergraduate participants. First, career unreadiness in terms of indecision and myth separately tended to cultivate anxiety. Second, authoritarian parenting appeared to foment career unreadiness in terms of career indecision and myth. Third, authoritarian parenting displayed a positive effect on anxiety. This effect partly went through a path mediated by career unreadiness, that is involving career indecision and myth. Overall, these findings manifest the harmful impact of career unreadiness and its roots in authoritarian parenting. The manifestation is justifiable with reference to behavioural inhibition theory (McNaughton & Gray, 2000; Neal et al., 2002). Accordingly, career unreadiness registers inadequacy in exploration and development due to behavioural inhibition, as triggered by authoritarian parenting. The theory also maintains that authoritarian parenting directly and indirectly breeds anxiety along with the inception of the behavioural inhibition system, which makes an individual physiologically and emotionally vigilant. Behavioural inhibition in career exploration, culminating in career unreadiness, is thus a mediating process that transmits the impact of authoritarian parenting on anxiety.

### Limitations and further research

Findings from this study are not yet conclusive, given the current limitations inherent in the study design. In particular, the cross-sectional design is limited to self-report and retrospective measures by undergraduates in a single university site in Hong Kong. Accordingly, the undergraduate reported authoritarian parenting during childhood, career unreadiness during study, and current anxiety. Given the limitations, the findings at best are suggestive of influences from authoritarian parenting to career unreadiness and anxiety, successively. The findings need corroboration, which requires further research using a more rigorous design to uphold the validity of its findings. Notably, such a design needs to ensure the temporal sequence of factors, typically by repeated measurement. At this juncture, further study can control for prior or trait anxiety to clarify the net effect of career unreadiness on state anxiety. The control is important to circumvent confounding by trait anxiety, which is missing in this study. Contextual variation is also important in further research to make it an explicit control and moderating factor. This implies the need for collecting data from multiple contexts to examine the contextual variation. In addition, further research will need to employ various modes of measurement to minimise errors due to the common source and method.

Another recommendation for further research includes making the causal mechanism transparent. Essentially, evidence for the mechanism based on behavioural inhibition theory has not been clear enough. Elucidation of the mechanism would require the identification of personal and environmental forces concerning the career, authoritarian parenting, and gender, which allow for conformity, arousal, resistance, and conflict. A challenge for further research is to illustrate a young person's operational behavioural inhibition system in relation to authoritarian parenting, career unreadiness, and anxiety levels.

The study also notes the compatibility between behavioural inhibition theory and social cognitive theory in explicating career unreadiness and its impact. As such, further research can seek to integrate the two theories by demonstrating the nexus between behavioural inhibition and self-efficacy belief. A nexus may happen when behavioural inhibition stemming from authoritarian parenting impedes self-efficacy belief involved in career readiness and exploration. Furthermore, another nexus may occur when self-efficacy belief in turn relieves behavioural inhibition about career exploration that fosters career readiness.

### Implications

In support of existing knowledge, behavioural inhibition theory is vital to defining the young person's career development and growth (Asendorpf, Denissen, & van Aken, 2008; Muris, van Brakel, Arntz, & Schouten, 2011). It explains relevant connections among authoritarian parenting, career unreadiness, anxiety, and their effects on both genders. Its implications for counsellors and other professionals in preventing anxiety in the young person are as follows. First, in reducing the young person's career unreadiness, notably career indecision and myth, fulfilling the requirement of the environment, including the university and society, is necessary. Second, neutralising the constraint and demand of authoritarian parenting requires alternative support for the young person's autonomy, including that in career development. Third, diminishing the career myth in the young person due to authoritarian parenting needs the provision of alternative support that orients her or him to meet realistic career and other environmental requirements. Fourth, reducing authoritarian parenting needs to rely on social or societal influence, as it is a cultural product (Lai et al., 2000). As a whole, reducing the constraining effect of authoritarian parenting and fostering the young person's independence and autonomy are consistent with alternative advice for boosting the young person's career development (Tokar et al., 2003). More importantly, such autonomy will enable the young person to meet realistic societal demands (Stoltz, Wolff, & McClelland, 2011). To this end, the guidance would need to start with a clarification of what is required of them to fulfil their function as youth. In all, the findings warrant efforts at enhancing students' career readiness during university education (Killgore, 2009).

In addition, the findings would have implications for managers, employees, and parents in enhancing career development. First, managers need to realise that the career unreadiness of university students and thus fresh graduates would make them anxious. Moreover, employees are likely to develop their careers in employment rather than in school or university (Sullivan & Baruch, 2009). Managers would therefore need to help employees who are fresh graduates to strengthen career readiness, through training, socialising, and/or mentoring. Similarly, employees who are fresh graduates would need to improve their career readiness in employment. They would also need to avoid the adverse impact of career unreadiness on their anxiety. That is, they can regard career unreadiness as a challenge in their career exploration and development and make them capable of resisting anxiety (Lent et al., 2000; Sullivan & Baruch, 2009). Third, parents would need to avoid authoritarian parenting, especially its adverse impacts on their offspring's career unreadiness and anxiety. Specifically, parents can help their offspring become autonomous in career exploration and as such free of behavioural inhibition. That is, parents can encourage their offspring to be active in dealing with the career and anxiety.
